# Pancreatic **β** cell function versus insulin resistance: application of the hyperbolic law of glucose tolerance

**DOI:** 10.1172/JCI176738

**Published:** 2024-02-01

**Authors:** Richard N. Bergman

**Affiliations:** Diabetes and Obesity Research Institute, Cedars-Sinai Medical Center, Los Angeles, California, USA.

The isolation of insulin from the pancreas by Banting and Best (1) and their colleagues was one of the great accomplishments of modern medicine, leading to a life-saving treatment for individuals with diabetes. Despite the discovery of a putative cure for diabetes, mysteries remained regarding the underlying pathophysiology. From observation it became clear that diabetes was not a single syndrome. Two kinds of diabetes were identified — the severe, juvenile-onset form of the disease, and an additional milder, adult-onset form of the disease. Himsworth was among the first to show that the mild form was associated with resistance to insulin administration (“insulin resistance”) (2). The elucidation of the radioimmunoassay confirmed that while insulin levels in the blood were vanishingly small in juvenile-onset diabetes, the milder adult-onset diabetes was associated with elevated insulin, supporting the idea that insulin resistance exists, particularly in the patients with obesity (3). Thus, at the dawn of the 20th century, researchers grappled with understanding the mechanisms underlying these two forms of the disease.

It soon became clear that, in juvenile diabetes (now called type 1 diabetes), circulating insulin levels were low owing to autoimmune destruction of the pancreatic β cells. However, the pathophysiology of the mild, but more prevalent, form, type 2 diabetes (T2D), was unclear. David Kipnis and Daniel Porte Jr. strongly supported the role of reduced β cell function as a primary pathogenic factor of T2D (4, 5). Clear evidence existed that the response of the β cells to glucose stimulation was reduced in those with T2D based upon accurate methods for measuring insulin. The question arose of how were those with T2D able to regulate their glucose even in the face of a severe pancreatic β cell dysfunction?

A second camp emerged, led to a great extent by Gerald Reaven, who argued that T2D was not so much due to insulinopenia, but to the inability of insulin to act on certain tissues — primarily liver and skeletal muscle (6). There was a critical need to measure insulin action to establish whether insulin resistance was a causal factor in T2D. One method to make such a measurement was the so-called “glucose clamp” (7). Reaven himself introduced a different method of measuring insulin sensitivity — the so-called “pancreatic suppression test” (8). This test involved the infusion of glucose and insulin into patients as well as a medicine to suppress endogenous insulin release from the pancreatic β cells. Under this infusion regimen, the ultimate resulting glucose level was interpreted as reflecting insulin resistance — clearly, if the resulting glucose level were high, one could conclude that insulin resistance was present. However, the glucose level per se was a qualitative, rather than a quantitative, index of insulin resistance.

The glucose clamp method introduced by Andres and colleagues was popularized by Ralph DeFronzo (9), who clarified the overall methodology of the clamp, including the use of radioactive tracers to measure glucose turnover in the body. It was possible to quantify the effects of insulin to increase glucose utilization (mostly by skeletal muscle) and to suppress endogenous glucose output from the liver and kidneys via lowering gluconeogenesis and glycogen breakdown.

A debate emerged as to the primary pathogenetic defect of T2D: was it insulin resistance or suppressed insulin release from the pancreatic β cell? This debate provided the framework for the *JCI* article that established the disposition index (DI) (10).

## A breakthrough discovery

Endocrine systems are “closed-loop” systems, wherein a hormonal signal generates a response that mitigates the signal. The regulation of the blood sugar also operates in a closed-loop manner: elevated glycemia is counteracted by a feedback signal — released insulin —which acts in turn to mitigate the glycemic stimulus by suppressing glucose production and enhancing glucose disposal.

Utilizing engineering technology, and in collaboration with Claudio Cobelli of the University of Padova, we developed mathematical models of insulin secretion and insulin action (11). The underlying purpose was to use these models, along with experimental or clinical data, to address the primary pathogenetic defect of T2D. We introduced the frequently sampled intravenous glucose tolerance test. The test involved intravenous glucose injection followed by intravenous insulin to generate stereotypical patterns of plasma insulin and glucose. The mathematical models were termed the “minimal models,” and all important regulatory parameters could be estimated from a single test. We were then able to exploit the combination of measured data (plasma glucose and insulin) and the models to address the pathogenesis of T2D.

## The hyperbolic curve

**Figure 1:**
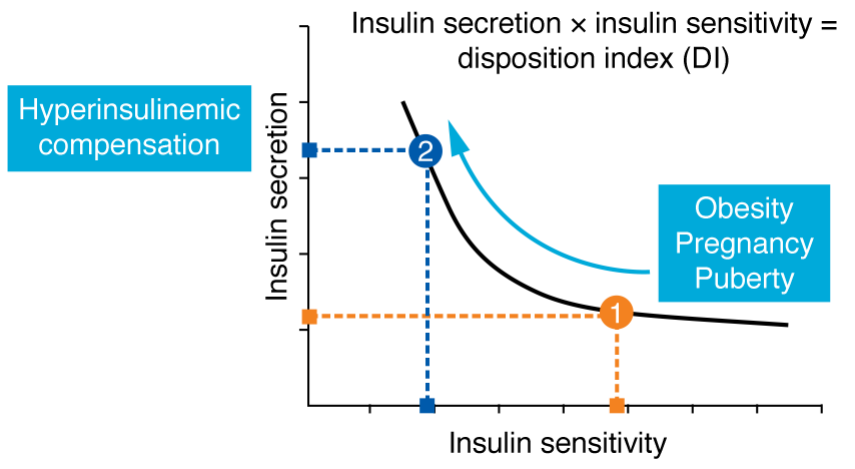
The hyperbolic law of glucose tolerance. Insulin resistance (in the face of, for example, obesity, pregnancy, or puberty) is represented by moving up and to the left on the curve (from position 1 to position 2). A higher position on the curve reflects enhanced insulin release from the pancreatic β cells. Improved insulin sensitivity (e.g., exercise) is represented by moving down and to the right on the curve, eliciting a lesser insulin response.

Remembering that these models were “closed loop” from a single test in one individual, we were able to calculate the insulin sensitivity index (SI), pancreatic β cell response, glucose effectiveness, and the degradation of insulin from plasma (10). We focused on insulin response (β cell insulin release) and SI because we assumed that they would be critical elements of how the glucose level was regulated. Under insulin-resistant conditions, e.g., in individuals with obesity, pregnancy, or during puberty, insulin sensitivity would be reduced. We expected that under such insulin-resistant conditions, the β cells would respond with enhanced insulin release, thus providing adequate blood sugar regulation (Figure 1).

There are times in scientific life when it is necessary to make a guess about how things work. Of course, we knew that the insulin response would increase in the face of reduced insulin action. But what hypothesized relationship could describe this pancreatic/extrapancreatic interaction? My intuition came into play here. I guessed that the relationship between insulin release and insulin action could be described by a rectangular hyperbola (Figure 1). I chose this function because it can be described by a single parameter — in the form of “*x* times *y* equals a constant.” In the context of carbohydrate metabolism, we proposed that insulin release × insulin sensitivity = constant. We termed the constant the DI.

## Interpreting the DI

What is the significance of the DI? Is it a stable value for individuals, or can it change over time? While the index could be interpreted as the ability of the insulin-sensitive tissues to respond to changes in ambient insulin in the blood (i.e., SI responds to changes in insulin secretion), it has most often been interpreted as the response of the β cells of the pancreas to changes in insulin sensitivity (12). Thus, in a normal individual, in response to a reduction in insulin sensitivity (obesity, pregnancy, puberty), the β cells will compensate with greater insulin response — movement “up the curve” (Figure 1). Alternatively, enhanced insulin sensitivity (e.g., due to exercise) would result in an individual moving “down the curve” — meaning that they would have a lower insulin response. The hyperbolic curve can then be thought of as an expression of the ability of the organism to *compensate* for environmental or physiological changes in insulin sensitivity. Thus, many investigators have interpreted the hyperbolic curve as an expression of the ability of the β cells of the pancreas to increase or reduce insulin release in the face of changes in insulin sensitivity (insulin action of tissues) (13). The DI has been interpreted as a measure of β cell function, i.e., β cell health. In this sense, the DI can be distinguished from other indices of β cell function (e.g., integrated plasma insulin during the oral glucose tolerance test), in that those static measures do not necessarily represent the ability of the β cells to *compensate* for changes in insulin action. This unique characteristic of the DI is responsible for its application to describe the ability of the pancreatic β cells to compensate for changes in insulin sensitivity. I am quite proud of the body of work from my lab and my close colleagues, Marilyn Ader, Giovanni Pacini, and Morvarid Kabir, in particular, as the DI has been so useful in studying glucose regulation in experimental studies, in clinical studies, in population studies, and in genetic studies. In fact, it appears that individuals can be associated with their unique DI as a measure of their ability to regulate their blood glucose.

## Application of the hyperbolic law

One very important classic application of the DI can be attributed to Christian Weyer, Clifton Bogardus, and colleagues, who have studied the pathogenesis of T2D in the Pima nation in Arizona (14). Weyer et al. reported that they were able to describe a population of Pima Indians in terms of the DI curve. They also showed that a subgroup of Pima Indians without diabetes that had higher (i.e., healthier) DI values did not develop frank diabetes over a 5-year period. In contrast, a second subgroup with lower DI values was observed to have increased risk of conversion to T2D over a 5-year observation period. Thus, if the DI is an expression of β cell health, we may conclude from the Pima data that lower DI — latent dysfunction of the β cells — will predict decline in glucose tolerance and eventual onset of disease. In this sense then, the DI has been utilized as parameter that can account for β cell functionality and predict risk of onset of T2D over time. Kodama and colleagues have performed a meta-analysis of the DI in a variety of populations, confirming the hyperbolic nature of the curve, independent of ethnicity (15).

The hyperbolic law has thus survived for many years and has been applied in many clinical studies. There is good evidence from the FUSION study that a DI value is associated with a single participant, and the heritability of DI indicates that it is strongly inherited (16).

## Whoops — it isn’t all the DI

The hyperbolic curve is simply a hypothesis regarding how the blood glucose is regulated. It is apparently true that, in many populations, the insulin action/β cell secretion relationship is adequate to describe the steady-state relation between these variables and how they may change longitudinally. But we now realize that this relation is less than perfect, and, in our laboratory, we are examining some of these imperfections of the so-called hyperbolic “law.” Longitudinal studies have confirmed that, in the face of developing insulin resistance, insulin levels increase. However, it appears that insulin does not totally compensate for extreme insulin resistance — thus there is a measurable deviation from the hyperbolic relationship at very reduced SI values. This difference may reflect a saturation effect — a limit to the ability of the β cells to compensate adequately for greatly lower insulin sensitivity even in otherwise healthy individuals. An additional factor is the degradation of insulin — primarily by the liver. While we (17) and others (18) have carefully investigated insulin degradation (“insulin clearance”), the extent to which changes in insulin clearance, as opposed to β cell function, contribute to the shape of the hyperbolic curve and longitudinal changes in the SI-insulin relationship remains to be elucidated. Further work in the field is needed to clarify the relative importance of insulin secretory compensation versus hepatic insulin clearance to the hyperbolic curve and the DI itself.

An additional factor determining glucose tolerance which is not reflected in the hyperbolic curve is the so-called “glucose effectiveness.” It is clear that glucose per se, independent of a change in plasma insulin, has potent effects to alter glucose turnover. New modeling-based approaches to calculate glucose effectiveness have appeared.

## Cluster analysis

Based on the work of Groop and colleagues, it has been suggested that several forms of T2D can be identified by cluster analysis of large populations of patients (19). The DI contributes to this discussion, as the insulin resistance/secretion relationship is a parameter that can differentiate several forms of the disease. It is possible that alternative forms of treatment may be appropriate for differentiated forms of T2D. It is hoped, therefore, that the quantitative analysis of carbohydrate metabolism can help to detect several forms of diabetes and lead to more appropriate treatments through personalized medicine.

## Version 1 02/01/2024

Electronic publication

## References

[1] Banting FG, Best CH (1922). The internal secretion of the pancreas. Indian J Med Res.

[2] Himsworth HP (1949). The syndrome of diabetes mellitus and its causes. Lancet.

[3] Yalow RS, Berson SA (1960). Immunoassay of endogenous plasma insulin in man. J Clin Invest.

[4] Perley MJ, Kipnis DM (1967). Plasma insulin responses to oral and intravenous glucose: studies in normal and diabetic sujbjects. J Clin Invest.

[5] Pfeifer MA (1981). Insulin secretion in diabetes mellitus. Am J Med.

[6] Reaven GM (1988). Banting lecture 1988. Role of insulin resistance in human disease. Diabetes.

[8] Shen SW (1970). Comparison of impedance to insulin-mediated glucose uptake in normal subjects and in subjects with latent diabetes. J Clin Invest.

[9] DeFronzo RA (1979). Glucose clamp technique: a method for quantifying insulin secretion and resistance. Am J Physiol.

[10] Bergman RN (1981). Physiologic evaluation of factors controlling glucose tolerance in man: measurement of insulin sensitivity and beta-cell glucose sensitivity from the response to intravenous glucose. J Clin Invest.

[11] Bergman RN (1979). Quantitative estimation of insulin sensitivity. Am J Physiol.

[12] Stefanovski D (2011). Consistency of the disposition index in the face of diet induced insulin resistance: potential role of FFA. PLoS One.

[13] Bergman RN (2002). Accurate assessment of beta-cell function: the hyperbolic correction. Diabetes.

[14] Weyer C (2000). Long-term changes in insulin action and insulin secretion associated with gain, loss, regain and maintenance of body weight. Diabetologia.

[15] Kodama K (2013). Ethnic differences in the relationship between insulin sensitivity and insulin response: a systematic review and meta-analysis. Diabetes Care.

[16] Elbein SC (1999). Heritability of pancreatic beta-cell function among nondiabetic members of Caucasian familial type 2 diabetic kindreds. J Clin Endocrinol Metab.

[17] Bergman RN (2019). Hypothesis: role of reduced hepatic insulin clearance in the pathogenesis of type 2 diabetes. Diabetes.

[18] Najjar SM (2002). Regulation of insulin action by CEACAM1. Trends Endocrinol Metab.

[19] Landgraf W (2022). Distribution and characteristics of newly-defined subgroups of type 2 diabetes in randomised clinical trials: Post hoc cluster assignment analysis of over 12,000 study participants. Diabetes Res Clin Pract.

